# Parents' experiences of psychotherapeutic support on the neonatal unit: A mixed methods systematic review to inform intervention development for a multicultural population

**DOI:** 10.1111/nicc.13194

**Published:** 2024-10-28

**Authors:** Kirsty Jane, Dean Wood, Katie Gallagher, Polly Livermore, Helen Shoemark, Glenn Robert

**Affiliations:** ^1^ Methodologies Division, Florence Nightingale Faculty of Nursing, Midwifery & Palliative Care King's College London Strand UK; ^2^ Hoslistic Family Services Noah's Ark Children's Hospice Barnet UK; ^3^ EGA Institute for Women's Health University College London London UK; ^4^ Orchid Office Level 3, Zayad Centre for Research London UK; ^5^ Boyer College of Music and Dance Temple University Philadelphia Pennsylvania USA; ^6^ Methodologies Division, Florence Nightingale Faculty of Nursing, Midwifery & Palliative Care King's College London London UK

**Keywords:** acceptability, multicultural, NICU, parents, psychotherapeutic intervention

## Abstract

**Background:**

Parents of infants admitted to neonatal intensive care require support to minimize the impact on their mental health and to encourage engagement with their infants to support infant neurodevelopment. Many interventions aim to address this need, but there is a lack of research considering the accessibility of these for a multicultural population.

**Aim:**

To systematically identify sources of psychotherapeutic support available for parents with infants admitted to neonatal care (NNU, neonatal intensive care unit [NICU] and special care units), assess their accessibility and acceptability and identify challenges and facilitators.

**Study design:**

Six electronic databases with no restrictions on language or date were used to identify relevant studies following Preferred Items for Systematic Reviews and Meta‐analysis (PRISMA) guidelines. Publications were included in the review if they reviewed parent experience of an intervention actively in place to support parent experience during the neonatal unit stay. Any studies where the intervention's primary aim was infant focused, such as developmental care, were excluded. All publications were quality‐assessed using quality appraisal tools appropriate for their design type. Data were extracted line by line using Sekhon et al.'s theoretical acceptability framework and questionnaire.

**Results:**

A total of 3309 studies were found, of which 36 studies met the inclusion criteria. Included studies were published worldwide between 2000 and 2023 and explored 15 different interventions. Challenges for parental engagement were due to preconceived ideas about intervention requirements and parents' ability to participate in them. Timely information and providers' experience in delivering the intervention were reported to support engagement and as being valuable for enhancing participant knowledge. The emotional content of interventions was found to be challenging by parents across most studies. This was prominent in interventions designed to be carried out in a group format and where keepsakes were created. However, the value of these interventions was in reducing parents' feelings of isolation through increased social support and providing a starting point for conversations with wider family and friends about the family's neonatal experience. Participant demographics were poorly reported, with only two studies taking into consideration the ethicality of the intervention.

**Conclusion:**

Poor reporting of participant demographics, and a focus on mothers as participants, means findings are not transferrable to the wider population of parents in neonatal units. Future studies should consider how to ensure that research and interventions are accessible to multicultural populations to improve the understanding of the acceptability of interventions. Better knowledge of neonates and the NNU setting amongst intervention providers could increase the accessibility of psychotherapeutic support for parents. Training for providers on how to manage sensitive conversations may also be beneficial to support parents during interventions.

**Relevance to Clinical practice:**

The impact of neonatal admission on parental mental health is increasingly recognized and reported. Interventions have been developed to reduce the negative impact on the mental health of parents. There continue to be significant health inequalities as a result of many services not taking into account the acceptability and accessibility of interventions in this setting for their multicultural populations. This review highlights the need for better reporting of participant demographics in research and the inclusion of those seldom heard to ensure interventions are culturally, religiously and linguistically appropriate for multicultural populations.

**What is known about the topic**

- Having an infant admitted to neonatal intensive care has a significant impact on parental mental health.
- This can have a negative effect on the infant's development and parents' ability to bond with their infant.
- Interventions are currently available on the neonatal unit to support both parents and infants and minimize these effects.

**What this paper adds**

- The ethicality of interventions and accessibility of them to people from a variety of cultures is rarely considered in research.
- Accurate recording of participant demographics and inclusion of participants representative of the diverse neonatal parent population is vital to accurately assess the acceptability and accessibility of interventions.

## 1 INTRODUCTION

Having an infant admitted to a neonatal unit (NNU) can have a significant impact on parents' mental health.^1^, ^2^ This can lessen their ability to engage and connect with their infant.^3^ Many parents will continue to present with symptoms of post‐traumatic stress (PTSD) up to a year after the discharge of their infant from the NNU.^4^ These symptoms can impact the whole family, including a negative impact on the long‐term neurodevelopment of the infant.^5^ This is especially significant for infants who are already at high risk of adverse developmental outcomes because of their neonatal admission.^6^, ^7^ As such, there is a clear need to support parents during their time on the NNU.^4^ We refer to the NNU as including the neonatal intensive care unit (NICU), high dependency and special care baby units unless specified.

## 2 JUSTIFICATION FOR REVIEW

Interventions have been designed to improve outcomes of parent mental health,^8^ bonding and attachment^9^ and infant development^10^, ^11^ whilst the infant is receiving care on the NNU. Evidence highlights that some of these interventions improve parent mental health^12^; however, intervention design often focuses on the parent at the cot side rather than considering the baby's cultural background and how to connect both parents and baby to their wider support network, particularly grandparents, aunts and uncles. As parents' wider social support often has a significant impact on their well‐being, this results in multiple interventions being required for parents to be fully supported, for example in the form of support groups as well as individual therapy and parent education.

Similarly, theories of attachment and bonding have been developed from Western, educated, industrialized, rich and democratic (WEIRD) country social structures and parameters, with studies excluding or labelling cultures which do not fit the model of a parent attachment figure and relative dependence on them.^13^, ^14^, ^15^ The need to change approaches within psychotherapeutic support to acknowledge diversity in cultures is gradually occurring.^16^ Understanding the accessibility of an intervention is a key component of the Medical Research Council's best practice guidance when designing and evaluating complex interventions.^17^, ^18^ There continues to be a disparity between White and Ethnic minority women accessing specific aspects of care and advice, likely due to cultural and language barriers.^19^ The Black ethnic group (including all subcategories) has the highest infant mortality rate in the United Kingdom with babies with an Asian ethnicity having the second highest rate.^20^ Interventions must therefore meet the needs of people from diverse backgrounds by being culturally sensitive and accessible to all.

## 3 AIMS AND OBJECTIVES

This review aimed to identify sources of psychotherapeutic support available for parents with infants admitted to neonatal intensive care and assess their acceptability and accessibility from a multicultural perspective.

## 4 METHODOLOGY

### 4.1 Information sources and search terms

Comprehensives searches of six databases were conducted by the lead researcher: MEDLINE, Embase, AMED, Psych INFO, CINAHL and Web of Science. All studies published before 5 January 2024 were included without restrictions on date or language. Searches were carried out a second time in March 2024 before analysis and then a final time before submitting to publication in May 2024.

Search terms were combined into three categories: neonatal intensive care (site), parents (population) and psychotherapeutic support (intervention). Three sets of heading terms were used for searches:

- neonatal intensive care unit, neonatal palliative care and special care neonatal unit;
- mother, father and parent; and
- psychotherapy, group psychotherapy, psychoanalytic therapy, counselling, psychodynamic psychotherapy, cognitive behavioural therapy, creative arts therapy, guided self‐help, mentalization‐based therapy, eye movement desensitization and reprocessing, mindfulness, holistic health, animal assisted therapy, family therapy, social support and pastoral care.

Heading terms were linked by ‘OR’ and searched with other categories using ‘AND’ (App 1).

The review protocol was registered with PROSPERO (CRD42023491268) and was reported in accordance with Preferred Items for Systematic Reviews and Meta‐analysis (PRISMA) ** guidelines^21^ (see App 5).

### 4.2 Selection process

Titles and abstracts from all searches were uploaded to the Rayyan software^22^ and screened independently by the first and second reviewers based on inclusion criteria, with blinding on (Table 1). Each reviewer allocated all the articles to either ‘Included’, ‘Excluded’ or ‘Maybe’. Blinding was then removed, and all conflicting and ‘maybe’ decisions discussed. Full texts of included articles were retrieved and reviewed by the lead author. Decisions to exclude were then further reviewed by the wider research team to finalize texts to include.

**TABLE 1:** Inclusion criteria.

Population	Parents with infants admitted to the neonatal unit
Intervention	Any form of psychotherapeutic support
Comparator	Standard neonatal care
Outcome	Acceptability and accessibility

Publications were included if they reported on parent experience of an intervention targeting support for parent experience during the neonatal stay. Studies where the primary aim of the intervention was infant focused, such as developmental care, were excluded. Breastfeeding support was also excluded as whilst the support may result in improved parental well‐being, this is not the primary aim of the intervention. Interventions could be provided by any health care professional or parent with no restriction on duration.

### 4.3 Quality appraisal

The quality of the included studies was assessed by the first reviewer using tools applicable to the study design (see App 4). These included the following: the Cochrane risk of bias tool (RoB2)^23^ for quantitative randomized controlled trials; the ROBINS‐I^24^ tool for non‐randomized studies of interventions (containing six aspects of risk of bias rated low to high); Critical Appraisal checklists (CASP checklists)^25^ for qualitative and cohort studies (10 aspects of quality appraisal and overall transferability of study); and the JBI checklist^26^ for case studies (eight aspects of quality review rated yes, no or unclear). Publication bias was not undertaken.

### 4.4 Analysis

**FIGURE 1:**
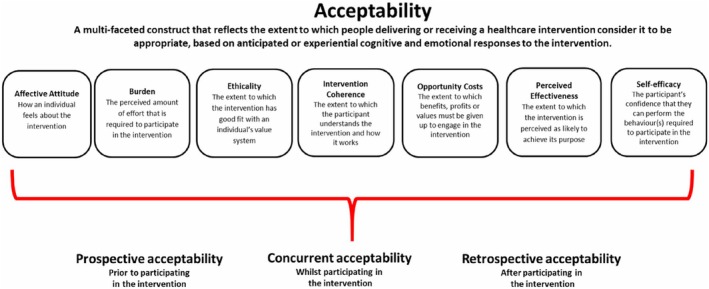
Graphical representation of domains of acceptability, Sekhon et al.^27^

Study characteristics (see App 2) were extracted initially and recorded as shown in Table 2. All articles were then coded line by line by the first reviewer, including quantitative data based on Sekhon et al.'s theoretical acceptability framework^27^ and questionnaire.^28^ This framework gathers participants’ prospective, concurrent and retrospective experiences to assess the acceptability of health care interventions. The framework consists of seven domains (see Figure 1) and is designed to consider reasons for discontinuation in studies as well as for lack of engagement with interventions. Questionnaire items were used to extract qualitative and quantitative data from the studies and allocate them to the relevant domains (see Table 2); for a table of analysis, see App 3.

**TABLE 2:** Graphical representation of data synthesis process.

Data analysis	Domain	Quantitative data	Qualitative data
	Affective Attitude	Frequency of use of services in relation to prior use/knowledge Preconceived ideas of intervention	Perceived areas of influence on participation Gender roles Recommendations for future practice Parent reported pre‐intervention anxiety (qual)
Domains of acceptability	Burden	Distance from hospital Inclusion criteria: access to car	Participant experiences of taking part in the intervention Recruitment challenges
	Ethicality	Reporting of age, ethnicity, religion, gender	Experiences of cultural matching
	Intervention Coherence		Supportive resources for understanding intervention
	Perceived Effectiveness	Reported results from quantitative outcomes measured	Parents reported effect and observations of the intervention on themselves and infant
	Opportunity cost	Number of live births Number of children at home	Family sacrifices
	Self‐Efficacy		Parents experience of participation and their ability to carry out the intervention
Study characteristics	Publishing data	Year of publication Country of Site	
	Intervention data	Intervention type Intervention provider	
	Research data	Number of participants Participant demographics Outcomes measured Inclusion/exclusion criteria	

Initial data categorized into domains of acceptability or study characteristics. Quantitative and qualitative data then extracted as shown.

From these domains, the accessibility and acceptability of the intervention as well as facilitators and barriers that impact parent experiences were identified and discussed by the review team. In addition, the demographic data of parents and the diagnosis of their infant were analysed to help contextualize their experiences of the interventions (see Table 2). Prevalent themes from gender subgroups were then identified.

## 5 RESULTS

**FIGURE 2:**
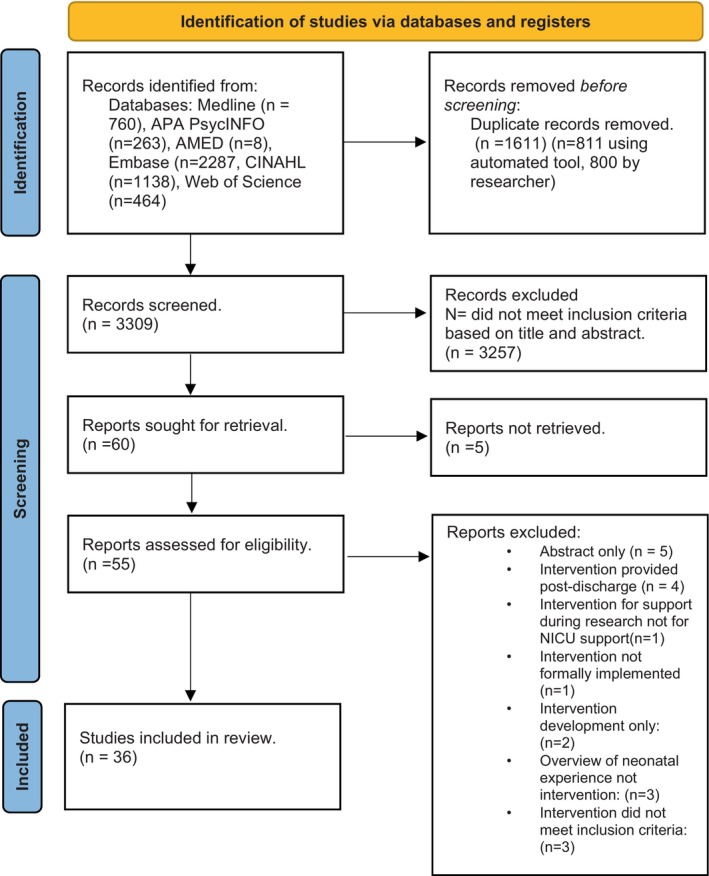
PRISMA flow chart.

The initial search of databases identified 3309 articles. After screening abstracts and titles, decisions were jointly discussed. These included 17 conflicting initial decisions of which 5 continued to be ‘Maybes’. Articles labelled ‘Maybe’ were retrieved and then discussed with the wider study team. After discussion, 60 articles were considered to meet inclusion criteria based on abstract and title alone and were retrieved for further screening. After reviewing full‐text publications, 36 studies were identified for the final review. The PRISMA chart detailing the search results and screening process is presented in Figure 2.

Studies were published globally between 2000 and 2023 with the majority between 2018 and 2023 (*n* = 21) and included 21 qualitative studies (*n* = 3 case studies), six quantitative studies, seven mixed methods, one data linkage study and one RCT (see Table 3). Studies included 15 interventions delivered mainly by professionals with specialist training except for two studies, which were provided by veteran NICU parents, journalling that had no formal support, one intervention that was provided by artists with no experience of the neonatal unit and two mindfulness interventions, which were a digital resource (see App 2).

**TABLE 3:** Study characteristics and results.

Author	Year	Country	Intervention	Provider	Number of participants	Demographics recorded	Inclusion/Exclusion criteria	Study design	Outcome measurements	Results
Aftyka et al.^29^	2003	Poland	Digital imagery sharing	Nursing staff	17 parents (4 males/ 13 females)	Gender Age	Recruited after stabilization of infant's health. Those with infants with unstable conditions were excluded.	Qualitative	Interviews with qualitative phenomenological analysis	Photographs and videos provided a useful communication tool. Parents’ initial response to first images were particularly emotional. Overall response: in‐person contact best but where not possible regular images in particular video supportive.
Archibald. S^30^	2019	UK	Father's support group	Female member of NICU psychology team and male member of chaplaincy	Forty fathers attended during study period. Average of four fathers per group.	None recorded	Fathers of infants on the NNU. No exclusion criteria	Qualitative	Feedback questionnaire combined with notes by facilitators from group	Main emerging themes: shared group experience, feelings of validation and an active family member.
Ardal et al.^31^	2011	Canada	Parent buddy matching by language/ culture	Veteran parents who attended a training programme. Unit social worker acted as a consultant for the buddy during the relationship.	8 mothers	Age Mothers' Parity Language spoken	Non‐English‐speaking mothers with infants who weighed <1500 g at birth and born at <30 weeks gestation	Qualitative	Semi‐structured interview with thematic analysis	Parent buddies helped non‐English‐speaking mothers ‘mobilize their strengths’. Culture and language matching particularly increased satisfaction. Linguistic matching increased access to services.
Beleninik et al.^32^	2019	Poland	Music therapy	Music Therapist	Four mothers, three fathers and six children (two single babies and four twins)	Age Education Economic status	Infants: born <35 weeks gestation age, medically stable. Parents: located within reasonable commuting distance, no physical or mental health problems, sufficient understanding and speaking of the respective national language.	Mixed‐methods feasibility study	PBQ: Parent Bonding Questionnaire ASQ‐3: Ages and Stages Questionnaire ASQ:SE‐2, Ages and Stages, Social Emotional Questionnaire EPDS: Edinburgh Postnatal Depression Scale GAD‐7: Generalized Anxiety Disorder Questionnaire PSS: Perceived Stress Scale MIBS: Mother Infant Bonding Scale Bayley Scales Interviews	Trial found to be feasible in Poland. Mothers were willing to participate in session; however, some preferred more passive engagement because of anxiety around singing in front of staff, therapist or other parents.
Bracht et al.^33^	2013	Canada	Parent education and support programme	Staff and Veteran Parents	Thirty‐nine mothers	Not reported	Mothers of infants born at <35 weeks gestation	Qualitative	Anecdotal feedback and formal evaluation via interview	Mothers were provided with tools to parent their infant and increase self‐confidence and emotionally prepare for discharge.
Corrigan et al.^34^	2022	USA	Music therapy	Music therapist	One hundred mothers enrolled, 44.3% completed the follow‐up	Age, ethnicity, marital status, gravida and parity	Legal guardian (age 18 +) of a preterm infant scheduled for an ROP examination on the NICU.	RCT with qualitative survey evaluation	Mother‐to‐Infant Bonding Scale Follow‐up survey with 5 mothers	44.3% completed MIBS follow‐up. No statistical difference between groups 1 week follow‐up. Themes of comfort, connecting family and personal growth.
Dahan et al.^35^	2021	Canada	Peer support group	Veteran NICU parents	Forty‐five parents (14 fathers, 31 mothers and 3 other family members)	Gender, relationship status, maternal age, number of other children in the family, distance from home to NNU by car.	Any parent in the NICU. Two parents had infants who died on the NICU; others included infants with complex medical requirements.	Longitudinal mixed methods evaluation questionnaire	Open‐ended questions in questionnaire and rating on a scale of 0–10.	95% reported meeting was useful. Overall evaluation 8.7/10. Themes: decreasing isolation (73%), hope and resilience (63%), practical parent information (32%), normalized emotions (92%).
Van Dokkum et al.^36^	2023	USA	Music therapy	Music therapist	Nine mothers and 1 father	None reported	Most parents had experienced critical situations with infant whilst on the NICU, 3 experienced their infant's death in the NICU.	Qualitative	Digital survey conducted through semi‐structured phone interviews.	Themes: memory‐making, connectedness, support of parental role, processing stressful NICU days. Participants recommended HeartSong as a viable NICU, accessible intervention.
Epstein et al.^37^	2023	Israel	Music therapy	Music therapist	Seven mothers	‘Parenting history’ (first time mother, etc.)	Infants: born <35 weeks gestation age, medically stable. Parents: located within reasonable commuting distance, no physical or mental health problems, sufficient understanding and speaking of the respective national language.	Qualitative	Semi‐structured interviews and interpersonal process recall. Interpretative phenomenological analysis.	Themes: Music therapy as a therapeutic haven; music therapy enabling integration of music into family relationship over time.
Ettenberger et al.^38^	2017	Columbia	Music therapy during kangaroo care	Music therapist	Thirty‐six participants (19 male)	Number of children, Age of mother	Infant inclusion criteria: infants born between 28 and 34 weeks gestation, birthweight < 2000 grams, kangaroo care initiated. Excluded: preterm infants who were medically instable, suffered from congenital malformations or if a surgery was planned for the week after the start of Music therapy.	Mixed‐methods study	Mother‐to‐Infant Bonding Scale, STAI S‐A (State‐Trait Anxiety Inventory) Immediate perspectives: semi‐structured interviews directly after therapy sessions Short‐term perspectives: questionnaires after last therapy session Medium‐term perspectives: In‐depth semi‐structured telephone interviews 3–5 months after hospitalization.	Statistically significant increased weight gain per day for infant during intervention period found of MT group (*p* = .036), length of hospitalization did not reach significance (*p* = .489). MIBS showed improvement but not statistically significant (mothers *p* = .806, fathers *p* = .128). STAI showed improvement with results for mothers being statistically significant (*p* = .007). Parents could relax, enjoy MT, session provided a distraction. Supported communication/ interaction, feeling connected Parents felt infants relaxed, parents felt empowered.
Feeley et al.^39^	2008	Canada	‘PMAC’ (Promoting Mother's Ability to Communicate) Programme: (a) how to identify and reduce anxiety and (b) education sessions on sensitive interaction	(a) Cognitive behaviour therapist (b) Graduate nurse and psychologist	Thirty‐three mothers	Age, education, country of origin, primary language spoken.	Inclusion: mothers of infant born weighing less than 1500 g, able to read English or French, lived within 1 hours drive. Excluded: mothers of infants with congenital anomaly, mothers who were not going to care for the infant.	Non‐randomized Quantitative feasibility study.	State‐Trait Anxiety Inventory, Parental Stress Scale: NICU, NICU parental beliefs scale, perinatal Post‐Traumatic Stress Disorder Questionnaire, Index of Parental Behaviour in the NICU. Participant questionnaire.	70% satisfied with programme; 30% mostly satisfied. 85% reported programme met almost or all their needs, 75% felt the number of sessions was right. Programme brochures easy to read (95%) and very helpful (75%). Most useful topics were preterm infant behaviour and appearance, infant communication cues and infant feeding behaviour.
Ghetti et al.^40^	2021	Norway	Music therapy	Music therapist	Three couples	Age Education level, Household economy, Relationship status.	Infants: born <35 weeks gestation age, medically stable. Parents: located within reasonable commuting distance, no physical or mental health problems, sufficient understanding and speaking of the respective national language.	Qualitative evaluation	Parent interview and questionnaire	Themes: parents of premature infants were willing to participate in music therapy where parental voice is the main means of interaction. Parents found noting infant interaction and responsiveness particularly interesting.
Grieb et al.^41^	2022	USA	Mindfulness	Video and audio recordings provided by researcher	Twenty‐six participants (15 intervention, 11 control)	Age, race/ethnicity, Insurance	Included: mothers of infants likely to be hospitalized for at least 2 weeks. Excluded if known to have serious psychopathology	Qualitative study	Semi‐structured, in‐depth interviews	Themes: mothers reported intervention helped ‘calm the chaos through recentring and fostering connections, find comfort in non‐judgemental acceptance, gain perspective on the situation and facilitate self‐care.
Haslbeck et al.^42^	2021	Switzerland	Music therapy	Music therapist	Six families (one x both parents, four mothers and one father)	Nationality Educational qualification	Parents of preterm infants <29 weeks, chronological age >7 days of life at the start of music therapy, clinically stable requiring no invasive cardiorespiratory support. Excluded if infants were diagnosed with a major congenital anomaly/ genetically defined syndrome, congenital malformation adversely affecting life expectancy/ neurodevelopment, admission for palliative care. Additionally excluded if considered to have insufficient knowledge of the German language.	Longitudinal qualitative study	Interviews conducted via phone or at home	Themes: positive impact of Creative Music therapy on infants, parents and bonding; parents were open‐minded to recommending the intervention as a complementary therapy; experience of overall healthy infant development despite unique developmental delays.
Helmer et al.^43^	2023	Sweden	Early collaborative intervention	Newborn Individualized Developmental Care and Assessment Program (NIDCAP) trained staff	Twenty‐three mothers	Age Parity Relationship status	Swedish‐speaking parents living in county of Ostergotland with infants born 30–36 weeks gestation without known major malformations or complications.	Qualitative study	Interviews with reflexive thematic analysis	Themes: Emotions raised by the intervention and how the intervention affected their parental role. Mothers' awareness of behaviour increased; increased ability to understand and communicate baby's needs. Intervention experienced as helpful for interaction with baby.
Hurst I.^44^	2006	USA	Parent support group, one‐to‐one support and telephone calls.	Veteran parent and nurse	Forty‐eight parents completed survey	Gender, age, ethnicity, occupation, marital status, insurance.	Able to access services in English	Data linkage	Survey of parental use and evaluation of services	78% utilized 1 support service exclusively. 18% utilized 2 formats concurrently. Continuity of case was report by parents as a critical component of the programme. Group support offered more opportunities for families to problem solve communication issues with staff and provided information that assisted parents in involvement in their babies' care.
Joyce et al.^45^	2021	Ireland	‘Beads of Courage’	Clinical staff trained in the programme	One hundred twenty‐three infants enrolled; 40 responses received from surveys	Not reported	Bereaved families excluded. Inclusion: Parents of infants born less than 32 weeks gestation	Qualitative descriptive survey	Survey	Parents found the programme extremely (72.5%)/helpful (27.5%); 85% felt the programme made the NICU stay easier.
Kobus et al.^46^	2021	Germany	Music therapy	Music therapist	Forty infants in intervention, 30 in control	Gender, age, education level, professional status, native language.	Excluded: birth in a different hospital from NICU, death, transfer to another hospital, cerebral haemorrhage, death of mother, insufficient knowledge of German.	Quantitative questionnaire, follow‐up of RCT	Likert‐style questionnaire at the time of discharge.	Positive change and enrichment during stay; parents were able to relax; parents could see the infant's reactions and felt they were calm and relaxed; 100% parents could imagine singing for their infant at home after discharge; 47% of parents stated they did not feel they developed a joy in singing.
Loewy et al.^47^	2021	USA	Music therapy	Music therapist	One family	None	Not stated	Case Study	Case study	Parents reported positive music therapy experience; value in having a recording
Marshall et al.^48^	2019	USA	Mindfulness	Staff trained in mindfulness‐based stress reduction	Thirty‐six parents completed mindfulness‐based training session, 28 finished study	Gender, race, distance from hospital, support, methods to cope with stress prior to study enrolment.	Inclusion: mothers and fathers of infants born <32 weeks gestation, Parents age 18 + . Excluded: Death of infant, Primary language other than English	Feasibility. Quantitative questionnaire with some open‐ended questions.	Perceived Stressor Scale‐NICU. Cognitive and Affective Mindfulness Scale‐R, 7‐item multiple choice questionnaire	79% reported mindfulness was helpful to cope with stress, 71% would continue practice after discharge. No difference in PSS:NICU or CAMS‐R at discharge. Harder to juggle time with responsibilities; challenging because parent wanted to focus attention on baby
Mendelson et al.^49^	2018	Austria	Mindfulness	Introduced by research assistant	Twenty‐four mothers	Race, education, relationship status, employment, experience with mindfulness/ meditation	English‐speaking and literate, if the infants were anticipated to require a NICU stay of at least 2 weeks following recruitment, infant was not experiencing acute medical crisis or imminent risk of death.	Pilot study: Quantitative evaluation	Patient Health Questionnaire 8 (PHQ‐8) Generalized Anxiety Disorder (GAD‐7) Stanford Acute Stress Reaction Questionnaire (SASRQ) Sleep Quality Index (PSQI) PSS‐NICU Mindfulness Questionnaire (FFMQ), Mother‐to‐Infant Bonding Scale (MIBS) Self‐Compassion Scale, Interviews	Significant improvements in depressive, anxiety and trauma symptoms, negative coping, NICU‐related stress and sleep (*p* < .05). Themes: perceived improvements in psychological distress and stress symptoms, self‐care and relationships.
Mouradian et al.^50^	2013	USA	Art therapy‐based scrapbooking group	Occupational therapist and art therapist assisted by social workers and a nurse	Forty parents	Age, Ethnicity/race, insurance, employment status, education level.	Included if parent at 18+ with an infant on the NICU.	Mixed methods study	State‐Trait Anxiety Inventory and brief interview.	Significant reduction in anxiety (reduction 12.7 points, *p* < .0001); participation offered distraction, relaxation; sense of hope; opportunity to share/ reduced isolation
Nottage. S^51^	2009	USA	Non‐medical support services (social worker, chaplain, family support group, sibling playgroup, family support centre, parent–parent support)	Multidisciplinary team	Forty‐four parents, 19 fathers and 25 mothers (26 families)	Gender Age, Language spoken, Ethnicity, Education level, Employment status, Household income	Inclusion: Parents with premature infants hospitalized in NICU age 18 years +. Singleton gestation. Infant hospitalized for minimum of 3 weeks. Able to speak and read English. Excluded: severe infant illness, imminent transfer/ discharge, anticipated death.	Quantitative questionnaire	Demographic questionnaire, Perceived Social Support Scale for Family and Friends (PSS:FA and PSS: Fr) and Utilization of Services Scale (USS)	Perceived social support from family or friends was not significantly correlated with use of support services (family *p* = .678, friends *p* = .911). Chaplaincy service second most used support service with 45% of participants using them without prior use for support. Parents knew about support but still did not use them.
Ormston et al.^52^	2022	UK	Music therapy	Music therapist	Mother and father	Not reported	Bereaved parents	Case study	Case study with parent feedback	Parents reported that the intervention provided time to focus on being a family; an escape from a medical environment; supportive at end of life and as a positive memory post‐death
Parker et al.^53^	2011	UK	Counselling/psychotherapy	Qualified counsellor/ psychotherapist in a sister role on the NICU	Six mothers	Not reported	Parents of infants who had accessed counselling whilst having an infant on the NICU between May 2007 and May 2009. Bereaved parents excluded.	Qualitative study	Interview	Themes: Counselling service viewed as a point of consistency and stability; unconditional psychological support. Felt to be vital that the counsellor had a working knowledge of neonates and NICU environment. A flexible approach was preferred.
Pearson et al.^54^	2000	USA	Parent sharing circle	Facilitator skilled in working with parents and families of premature infants	One hundred and four parents (59 mothers, 45 fathers)	Not reported	Included if parent had attended Parent Circle.	Qualitative programme evaluation	Evaluation questionnaire	Parents reported that the intervention helped them gain perspective on their situation, feel supported, learn developmental concepts, locate resources and optimize interactions with their infant.
Preyde M and Ardal. F^55^	2003	Canada	Parent buddying	Trained veteran parents of preterm infants	32 mothers	Age, Relationship status, Education, Annual income, Ethnicity	Excluded if infant died	Quantitative cohort study	PSS‐NICU State‐Trait Anxiety Inventory, Beck Depression Inventor, Multidimensional scale of perceived social support.	87.5% mothers indicated their buddy was very helpful or helpful, two mothers indicated no difference and one mother found them unhelpful. At the fourth week, the intervention group report less stress than those in the control group (*p* < .001), and at the 16th week the intervention group reported less state anxiety (*p* < .05), less depression (*p* < .01) and greater perceived social support (*p* < .01) than the control group.
Russel et al.^56^	2021	USA	Journalling with some prompts in journal	Journal provided whilst mother on postnatal ward	Ninety‐seven parents Control (*n* = 47): 66% female Intervention (*n* = 50): 72% Female	Age, Gender High school education Number of other children at home Prior NICU experience Social media use Mental health treatment	Mothers, fathers and legal guardians or infants admitted to NICU >28 weeks gestation. Anticipated at least 5 days admission. Fluent in English, Spanish or Arabic. Excluded: expected death of infant or discharge within 5 days of admission.	Randomized controlled pilot study. Mixed methods	Hospital Anxiety and Depression Scale (HADS), qualitative journal use data.	Significant decrease in anxiety from baseline, most prominently in fathers; 79% used the journal, 93% felt it was helpful, in particular for expressing and processing emotions; 41% felt it improved their overall NICU experience, most indicated a neutral answer.
Schwarz et al.^57^	2004	USA	‘Rush Keepsakes’ Scrapbooking group and family photo shoots.	NICU staff nurses	Not reported	Not reported	All parents with infants on the NICU	Case study of intervention development and evaluation	Case study with informal parent feedback	Parents reported appreciation of intervention. Sessions felt to provide respite from the NICU and opportunity to interact with other families.
Scott. Z and Archbald.S^58^	2020	UK	Fathers support group	Female member of NICU psychology team and male member of chaplaincy	Eight fathers (4 group, 4 individual)	Not reported	Fathers with infants admitted to the NICU	Qualitative analysis	Thematic analysis of group discussion	Themes: ‘ups and downs’ of NICU journey; juggling NICU and life outside; feelings seen, included and cared for; shared experience.
Shoemark. H^59^	2018	Australia	Music therapy informed parent education programme	Music therapist	Thirteen mothers	Age, education, ethnicity, First experience of parenting, music experience	Mothers with access to her infant for at least 3 days prior to recruitment, Excluded: known mental health issues, involvement from government agencies	Qualitative programme evaluation	Interviews	Participants reported the programme to be useful, most useful content reported to be about infant behaviour and cues for interactions. Weakest aspects of translation were for key language and some aspects of visual content in the booklet.
Simon et al.^60^	2021	USA	Cognitive behaviour therapy‐based intervention group	Psychology fellows and student therapists	Nineteen mothers	Age, race, education, income. Employment, marital status	English‐speaking mothers greater than 18 years of age of infants 23–34 weeks gestational age. Excluded: Mothers of infants awaiting cardiac surgery, those with congenital abnormalities, those who were unlikely to survive. Mothers evaluated to be at high psychiatric risk.	Quasi‐experimental intervention development and evaluation. Quantitative.	Beck Anxiety Inventory, Beck Depression Inventory Davidson Trauma Scale, Maternal Satisfaction Questionnaire (Likert)	Overall satisfaction mean score of 4.49, helpfulness score 4.57. Anxiety did not change in 6 weeks but reduced at the 6‐month follow‐up, and depression reduced significantly in 6 weeks (*p* = 0.043). Trauma scores at baseline were above PTSD cut‐off and were below at the 6‐week follow‐up.
Turner et al.^61^	2015	USA	Group parent support and education	Child and adolescent psychiatrist and neurodevelopmental physiotherapist	Sixteen mothers (3 men declined)	Not reported	Parents of infants at any stage of NICU stay who had attended at least one NICU parent support group, fluent in English.	Cross‐sectional qualitative study	Cross‐sectional interviews	Themes: Guilt related to pregnancy ending prematurely; anxiety about possible death of baby; positive feelings about the baby; anxiety about holding the baby; anxiety about parenting capabilities; the parent–nurse relationship; support group provided shared emotional experience.
De Vasconcelos et al.^62^	2008	Brazil	Mothers support group	Multidisciplinary team (nurses, social workers, psychologists and students in nursing, biology and social communication)	Sixteen mothers	Age	Women with preterm infants in the NICU for 7 days or more	Exploratory descriptive study	Interviews with guided questions	Themes: Support group provided a space to express themselves openly; facilitators felt to be there to listen; initially listening to others is highly; emotional; group provided social support; developed hope and strength; group was comforting for other family members to know that mothers were being supported; group provided a distraction from the environment, made it feel more relaxed and joyful; enjoyment of group activities; group developed sense of strength to continue.
Wharton et al.^63^	2023	USA	Group trauma‐focused cognitive behaviour therapy	Psychology fellows and student therapists	Nineteen mothers baseline assessment, Thirteen post‐treatment assessment Seven completed 6‐month follow‐up.	Not reported	Adult English‐speaking mothers of infants 24–34 weeks gestational age. Excluded: mothers of children awaiting cardiac surgery or unlikely to survive.	Pre/post quasi‐experimental study. Mixed methods.	Trauma narrative questionnaire, Distress Rating Qualitative open‐ended questions on their experiences writing and reading aloud their trauma narrative.	Mothers perceived the exercises as positive and helpful. some participants felt exposed when reading their narrative aloud (7/9 reported an increase in distress at these points); no participants regretting writing or sharing their narrative; participants felt less alone and proud of themselves; participants reported they did not have enough time to process their narrative; 7 participants reported distress levels by the end of the final session.
White et al.^64^	2010	UK	‘Art therapy’	Artist	Forty‐one family members participated: (25 mothers, 13 fathers, 3 siblings) 10 parents completed questionnaires	Not reported	Parents with ‘stable’ infants on the NICU	Cohort evaluation. Qualitative study	Feedback questionnaire	Parents who participated enjoyed the experience, claimed it relived their stress and wanted to continue. The project did not interfere with work of the unit.

### 5.1 Outcome measurements

All articles included in this review evaluated parents' experience of the intervention. Table 4 shows the outcomes measured and the measurement used. Most studies conducted interviews to assess parent experience (*n* = 17) and analysed this using thematic or interpretative phenomenological analysis. Two studies gathered feedback of acceptability and satisfaction via Likert scales. In addition to parent experience, the most predominant symptoms assessed to evaluate effectiveness of the interventions focused on anxiety (*n* = 7), parent stress (*n* = 5), depression (*n* = 4), trauma (*n* = 4) and bonding (*n* = 4).

**TABLE 4:** Outcomes and measures.

Outcome	Measurement	Total number of articles
Experience (all studies)	Feedback *n* = 3 Interview *n* = 17 Questionnaire *n* = 9 Survey = 7 Quantitative evaluation form (helpfulness/satisfaction/ acceptability) = 4 Qualitative analysis of group transcription = 2	36
Anxiety^38^, ^39^, ^49^, ^50^, ^55^, ^56^, ^60^	State Trait Anxiety Inventory, GAD‐7, Beck Anxiety Inventory, Patient Health Questionnaire	7
Stress^32^, ^39^, ^48^, ^49^, ^55^	Parental Stressor Scale (PSS‐NICU)	5
Depression^32^, ^55^, ^56^, ^60^	Beck Depression Inventory (BDI‐II), Edinburgh Postnatal Depression Scale (EPDS), HADS, PHQ‐8	4
Bonding^32^, ^34^, ^38^, ^49^	Mother‐to‐infant bonding scale	4
Trauma^39^, ^60^, ^63^	SASRQ, PTDS questionnaire, Trauma Narrative, Davidson Trauma Scale, Distress rating 0–10	3
Social support^51^, ^55^	Multidimensional scale of perceived social support	2
Mindfulness^48^, ^49^	FFMQ, Toronto Mindfulness scale, Cognitive and Affective Mindfulness scale	2
Satisfaction^35^, ^60^	Likert scale	2
Behaviour (parent)^39^	Index of parent behaviour	1
Beliefs^39^	Parenting belief scale	1
Coping skills	Brief COPE	1
Sleep quality^49^	PSQI	1
Self‐compassion^49^	Self‐compassion scale	1

### 5.2 Acceptability

Most studies included qualitative data on the parent experience of the intervention (*n* = 29). Using Sekhon et al.'s theoretical acceptability framework,^27^ these have been grouped into the seven domains shown in Figure 1.

#### 5.2.1 Affective attitude

Parents participating in counselling/ psychotherapy reported that their preconception of the intervention affected their willingness to engage.^53^ Perceived gender roles and expectations additionally affected engagement where fathers reported the intervention was more appropriate for mothers^40^ or declined to participate and asked that the mother be spoken to instead.^61^ In studies where both parents were invited to participate, a higher percentage of mothers did so (see Table 3). Initial anxiety towards participating due to entering the unknown was reported in a music therapy (MT) intervention.^42^ However, a different MT intervention reported this to be resolved if adequate information was provided prior to commencement.^36^ Such information provision was recommended by participants as a future implication from a longitudinal MT study.^40^ Parents highlighted the value of being told about available interventions in increasing attendance by encouraging parents' consideration of their own needs.^44^ In one MT intervention where parents participated either through being present with their baby or through active singing, parent's relationship with music was reported to influence how much they used music; those who had less experience with music in their lives perceived themselves as ‘less capable singers’.^40^ In contrast, in a study investigating the use of non‐medical services the chaplaincy service was reported to be the second most used service despite 45% of participants having no prior experience of engaging with a chaplain.^51^ A study where artist's visited parents at cot side reported value in its provision of equipment (digital camera), which was otherwise not affordable for the participants,^57^ suggesting this was an incentive that influenced participant engagement.

#### 5.2.2 Burden

Participants in two studies^48^, ^58^ reported challenges in engagement with the intervention because of conflicting feelings on using time for themselves rather than focusing on their baby. Fathers experienced feelings of conflicting responsibilities in dividing their time between focusing on their partner and the baby before themselves.^30^, ^58^ Participants of an early collaborative intervention reported the intervention as an ‘extra burden’^43^ initially. Five studies reported participants proximity to the hospital with four of these studies stating ‘access to a car’ or travel of 30–60 min in the inclusion criteria. Travel to the hospital was considered as a burden for families and a potential barrier to accessing the interventions provided.

#### 5.2.3 Ethicality

Only two studies considered the extent to which the intervention was a good fit with the individual's value system: one reflected on cultural matching of parent buddies^31^ and the other on the usefulness of supporting materials in regard to format and vocabulary.^59^ Other confounding factors on value systems such as age, gender, ethnicity and religion were not consistently reported (see Table 3 **)**.

#### 5.2.4 Intervention Coherence

Parents participating in an ‘early collaborative intervention’ where parents were supported in participating in the infant care by a trained NIDCAP member of staff reported that information provided by staff or in leaflet form supported their understanding of the intervention and how they could engaged with it.^43^ Similarly, participants in a MT‐informed parent education programme reported that images were supportive for educative purposes and increased understanding of the intervention.^59^

#### 5.2.5 Opportunity costs

The main cost experienced by participants was giving up time with their infant to attend the intervention.^57^ Parents in a study looking at their experiences of accessing a parent support group and support telephone calls felt they needed to choose between time for themselves or time for their infant.^44^ Studies included extremely premature infants and infants with complex medical needs and reported that an expectation of focus being removed from the infant was experienced as particularly difficult when the infant was unwell.^44^ Ten studies (see Table 3) reported mothers' parity (number of births to viable gestational age). However, only two of these reported how many children were at home.^30^, ^51^ The remaining studies did not consider the impact of parent's time split between hospital and home life or any other caring or sibling responsibilities.

#### 5.2.6 Perceived effectiveness

Parents identified interventions as reducing isolation, normalizing the environment and the experience of having a baby on the NNU, providing a source of hope and providing an escape from hospital life. Group interventions, whilst providing social support, were experienced as emotionally challenging because of being exposed to other parents' emotions, and in the intervention where parents read out loud their trauma narrative to the group, this feeling of exposure resulted in an increase in distress at these points.^63^ Fathers in groups exclusively for fathers felt that the provision of the group had acknowledged and given value to their part in the family.^30^, ^58^

As well as reducing isolation through social support, participants reported enjoying aspects of interventions that provided something to share with family outside the hospital. These interventions included heartbeat/song MT, scrapbooking, developmental support programmes, digital photography sharing and artist visits. However, whilst interventions such as ‘beads of courage’ provided a visual representation of the infant's journey,^45^ keepsakes were also experienced as (a) anxiety provoking^29^ when, for example, images were shared with parents during the COVID pandemic and (b) challenging with beads highlighting what a baby had lived through.^45^ This was particularly true for those with a critically ill infant and the emotional content of scrapbooks.^57^ In this context, it was also noted that images captured by artists of the NICU environment were as provocative as those of the baby.^64^

Interventions that promoted parent engagement with their infant were perceived to increase parents' sense of parental identity and confidence in parenting ability. For example, an increase in the value of their own voice and a development of belief that they were ‘doing enough’^59^ in a MT‐informed parent education programme.

Parents’ understanding,^59^ increase of focus^64^ and seeing a positive effect on infant^46^ through the interventions such as MT parent education, artist's imagery and MT where the parent was requested to be present were reported to improve the experience of the NNU and, in the early collaborative intervention, develop a sense of personality in the infant.^43^ In five studies, participants reported a feeling of increased closeness or bond to their infant, which also continued post death in two MT studies.^36^, ^52^

Artists' visits,^64^ mindfulness interventions^41^, ^48^ and MT^34^, ^36^, ^38^ where parents had either had recordings provided for their use or had the option of being present but not participating were reported to be of value for parents’ self‐care, providing time to focus on themselves and for relaxation.

#### 5.2.7 Self‐efficacy

Studies of MT interventions reported participants' feelings of inadequacy or low levels of ability to be able to carry out the intervention, including feelings of embarrassment in using their voice in front of professionals. It was not clear in studies whether this continued during the intervention or was solely prior to attempting carrying out the required tasks. The perception of being judged or exposed in interventions was reported in both MT and the ‘early collaborative intervention’.^43^ Positive feedback from the infant^32^ and the intervention provider^43^ was felt to reduce feelings of judgement and embarrassment.

### 5.3 Participant suggestions for future practice

Further points were raised as recommendations for improving future implementation of interventions. Reliability and trust in the provision of the intervention was a theme across several studies. Participants attending support groups or paired with veteran buddies nominated a preference for those facilitating or providing interventions to have knowledge in the neonatal experience and the diagnosis of the infant^31^, ^44^ and expertise in the method being delivered.^33^ In the fathers' group, participants reported appreciation of one of the facilitators being the same gender as themselves.^58^ Similarly, groups were felt to be more challenging when other parents' experiences were not relatable.^53^ Reliability of the provider^31^ and provision^58^ and continuity of the provider^44^ were reported as important and supportive factors. In the study provided by artists without prior experience of the NNU, artists' own anxieties about the environment were reported to impact their confidence in approaching parents.^64^

Activities in groups such as sharing food^58^ and creative activities^62^ were appreciated and felt to assist group dynamics. Providers of the scrapbooking intervention felt the activity provided an opportunity for ‘social support’ without expectation to listen to others,^57^ therefore lessening the emotional burden previously mentioned.

Recommendations for better provision of supporting information were also reported. These included requests for signposting to additional support relevant to their situation^53^, ^58^ or available on the unit.^44^ Resource signposting^54^ and topics that supported understanding on infant behaviour, appearance and communication^39^ were reported to be particularly useful. Whilst images were perceived to have potential to enhance education^33^ and to help break up blocks of text in booklets,^59^ in two MT studies it was suggested that videos or in‐person demonstrations were of higher value.^40^, ^59^

## 6 DISCUSSION

Our findings have been drawn from heterogeneous studies to assess the acceptability of sources of psychotherapeutic support. This has resulted in the inclusion of both quantitative and qualitative data from which to draw findings and conclusions. Whilst qualitative data provide understanding of participant experiences in a freely expressive context, the quantitative data have added to this through the provision of statistical evidence as a direct result of the interventions. The quantitative studies also demonstrate researcher areas of focus when evaluating and accessing the acceptability of interventions. The overall findings highlight the potential for current sources of psychotherapeutic support to provide parents with hope through increased social support and sense of parental identity. Findings also draw attention to challenges in engaging with interventions as well as facilitators to be considered for future intervention development and delivery. The reporting of participant demographics was very limited.

### 6.1 Accessibility and acceptability to a multicultural population

Psychotherapies are dominated by a largely White population of professionals and are often deemed to be targeted at people from more affluent socio‐economic backgrounds. This can make them unattractive or potentially inaccessible to those who may benefit from mental health services the most.^65^ The values behind psychotherapy practice are often focused on individual agency, which is not necessarily appropriate for people from non‐Western cultures. Most of the studies included in this review did not report participants' ethnicity or social support and did not take this into consideration when evaluating the acceptability of the intervention. Only one study focused on the adjustments of an intervention to meet cultural values in the form of matching parent buddies by language and culture.^31^ The perceived effectiveness of this intervention was largely connected to buddies being deemed relatable and understanding of cultural values as well as the neonatal experience.

Perceived power dynamics because of professional roles could be found across a number of studies including those implementing MT^34^, ^37^, ^40^ and where parents were observed as part of a parenting programme provided by a NIDCAP professional. Parents of infants on NNUs are likely to experience feelings of inadequacy and exclusion because of professionals needing to take the lead in their infant's care.^66^ Addressing power dynamics should be considered in interventions that aim to support parents. In contrast, groups led by veteran parents^31^, ^33^, ^35^, ^44^ and professionals of the same gender^30^, ^58^ as well as when participants had commonalities such as infant diagnosis were felt to be supportive because of increased accessibility and offering a source of hope from someone that was deemed to be relatable. Connection with staff and support services is additionally likely to reduce feelings of isolation experienced by parents of neonates, in particular those with complex diagnoses.^67^

### 6.2 Challenges

#### 6.2.1 Emotional impact

Interventions that included parents interacting with their infant were reported as initially challenging to engage with.^36^, ^40^, ^43^ Research shows that seeing their infant within a medical environment is a source of stress for parents,^68^ and therefore, parents may find prolonged focus on the infant and the infant's experience emotionally challenging. Similarly, reminders of the medical procedures and challenges that the infant had faced were also emotionally challenging.^45^ The heightened level of parental stress must be considered when implementing interventions. Providers may benefit from training enabling them to support parents emotionally to engage with interventions. Parents reported hands‐on demonstrations as potentially more valuable than images.^59^ Providers should consider signposting at the end of interventions to enable parents to continue to access support.

Participants, whilst finding similarities in experiences valuable, reported challenges in listening to other parent's emotions further demonstrating the emotional burden parents are experiencing on NNUs. With the possibility of social support outweighing some of the emotional burden of group work, facilitators should be aware of this emotional impact and ensure that participants are well supported. As noted by participants who scrapbooked as part of the support group,^57^ activities may provide a means of supporting parents regulate their engagement with group conversation.

#### 6.2.2 Managing responsibilities

There is limited research on the experiences of fathers with infants on the NNU.^69^ However, it is known that these experiences and strategies for coping differ from birthing parents.^70^ This review supports the view that fathers experience additional challenges in attending interventions because of concerns with looking after their partner as well as their infant.^30^, ^58^ Mothers also reported challenges in engagement with interventions because of demands on them as part of the care of the baby^44^, ^57^ and wanting to focus on the baby^48^ rather than themselves. Providers should consider providing interventions that are available outside traditional working hours and include childcare for siblings to support both parents in engaging. Flexibility of timing for the delivery of interventions could promote awareness of the ongoing challenges parents face and recognize the unpredictability of the environment and infant's neonatal journey.

### 6.3 Facilitators

#### 6.3.1 Provision of information

Provision of information was identified across several studies as a facilitator to increase accessibility and acceptability of an intervention. Interventions that may be unfamiliar to parents and involve engagement with the infant may require further explanation to reassure parents that no harm will come to their already fragile infant. Parents want to protect their infants at the start of their lives and particularly when they are aware of the potential death of their baby.^71^ With the provision of adequate information prior to the commencement of the intervention, parental anxiety is likely to be lessened because of improved understanding. This may address reported challenges with low self‐efficacy and wariness of MT studies,^40^ as MT is potentially perceived as a less evidence‐based practice because of its relative novelty in comparison with other allied health professions.^72^ Additionally, information may resolve any myths or misconceptions about interventions. Participants also reported the value of information after sessions for signposting to further relevant support available.^39^, ^58^ This signposting is likely to reduce feelings of isolation because of the reassurance for parents that they are not alone in their experience.

#### 6.3.2 Intervention providers

Several studies reported participants' appreciation of providers being knowledgeable and relatable. This came in two forms: firstly, the interventions were felt to be most successful when those delivering or recruiting for the intervention were perceived to be competent in understanding and delivering the intervention^33^ and secondly when those who were leading the intervention were perceived to be relatable and trustworthy.^30^, ^31^, ^35^, ^47^, ^55^, ^58^ It is possible that the perceived knowledge of providers is also a result of effective communication of information about the intervention. Currently, training requirements for professionals delivering arts therapies and mindfulness interventions on NNUs is variable. Whilst some neonatal specific training is in place for MTs, this training is not recognized within the United Kingdom and has a limited focus on preterm development or newborn behaviour. Art therapists and mindfulness coaches may have little knowledge of the NNU prior to the delivery of interventions. To best prepare providers for supporting parents on NNUs, further training may be beneficial within their professional qualification and registration.

## 7 LIMITATIONS

This study was based on articles retrieved via databases only. Further hand‐searching of journals and reference lists was not undertaken; there is a possibility that further articles not retrieved by the database searches may meet inclusion criteria. Because of poor reporting of participant demographics, this review was unable to assess the acceptability of interventions for people across cultures, particularly in connection with cultural values (including the experience of same sex parents). Additionally, the studies were heavily mother‐focused, and the findings are not transferrable to fathers of infants on NNUs. Only three studies included parents under the age of 21, with the exact numbers of this population unclear. It should be noted that three of the authors are qualified music therapists working collaboratively with sociologists and nursing professionals as part of the research team. These authors acknowledge their interest in creative therapy approaches. Whilst the research team experience has given a variety of views of the collected data, all researchers are driven to improve health care and address health inequalities.

## 8 RECOMMENDATIONS FOR FURTHER RESEARCH AND PRACTICE

Future assessment of the acceptability and accessibility of interventions should aim to ensure that the multicultural neonatal population is better represented in studies, and the ethicality and burden of any intervention is considered. Effective communication about the intervention and providers' knowledge of the environment can facilitate this. This review suggests attending to such issues would optimize the development of interventions that are multiculturally accessible and acceptable and support both parents' psychological needs.

## 9 CONCLUSION

There is a lack of consideration in the current literature relating to the appropriateness of psychotherapeutic interventions for parents whilst their baby is on a NNU, particularly in relation to (a) cultural, religious and linguistic appropriateness and (b) the needs of both parents. Better recording of participant demographics and ensuring research is accessible to a diverse participant group will improve outcomes and help address health inequalities as demonstrated in the MBRRACE report.^19^ Themes of emotional burden, knowledge of the intervention and the neonatal environment demonstrate a need for providers to be better trained in managing difficult conversations and understanding newborn behaviour and the NNU environment itself.

## FUNDING INFORMATION

This article was written as part of the lead authors Doctoral Clinical Academic Fellowship awarded in June 2023 (NIHR303074). Katie Gallagher (NIHR302193) and Polly Livermore (NIHR302864) are also sponsored by NIHR ICA awards.

## Supporting information


**Data S1.** Supporting Information.

## Data Availability

The data that supports the findings of this study are available in the supplementary material of this article.
