# Supplementary material for: Parents' experiences of psychotherapeutic support on the neonatal unit: A mixed methods systematic review to inform intervention development for a multicultural population
Source: Nurs Crit Care. 2024 Oct 28;30(3):e13194. doi: 10.1111/nicc.13194 (PMC12096267; doi:10.1111/nicc.13194)
Supplement: Supplementary file 1 — Data S1. Supporting Information. [file NICC-30-0-s001.docx]

**App. 1 Search terms**

| **Database** | **Search Strategy** |
| --- | --- |
| **Medline** | 1 exp Intensive Care Units, Neonatal/  2 "neonatal intensive care".mp  3 NICU.mp.  4 "neonatal palliative care".mp.  5 1 or 2 or 3 or 4  6 exp Parents/  7 parent*.mp.  8 exp Mothers/  9 mother*.mp.  10 exp Fathers/  11 Father*.  12 6 or 7 or 8 or 9 or 10 or 11  13 exp Psychotherapy  14 psychotherapy.mp.  15 exp Psychotherapy, Group/  16 group psychotherapy.mp.  17 exp Psychoanalytic Therapy/  18 psychoanalytic therapy.mp.  19 exp Counselling/  20 counselling.mp.  21 psychodynamic therapy.mp  22 exp Psychotherapy, Psychodynamic/ 807  23 exp Cognitive Behavioral Therapy/ 37400  24 cognitive behaviour* therapy.mp  25 exp Art Therapy/ 1748  26 "creative therap*".mp  27 "guided self help".mp  28 exp Mentalization-Based Therapy/ 21  29 mentalization based therapy.mp  30 "mentalization based therapy".mp.  31 eye movement desensitization reprocessing.mp  32 exp Eye Movement Desensitization Reprocessing/ 396  33 exp Mindfulness/  34 mindfulness.mp.  35 exp Holistic Health/  36 holistic health.mp  37 exp Animal Assisted Therapy/  38 animal assisted therapy.mp  39 family therapy.mp  40 exp Family Therapy/  41 exp Social Support/  42 social support.mp.  43 peer support.mp.  44 exp Pastoral Care/  45 pastoral care.mp.  46 13 or 14 or 15 or 16 or 17 or 18 or 19 or 20 or 21 or 22 or 23 or 24 or 25 or 26 or 27 or 28 or 29 or 30 or 31 or 32 or 33 or 34 or 35 or 36 or 37 or 38 or 39 or 40 or 41 or 42 or 43 or 44 or 45 476342  47 5 and 12 and 46 |

**App. 2 Intervention Characteristics**

| **Author** | **Intervention** | **Provider** | **Psychotherapeutic Aim of intervention** | **Group/ individual** | **Infant included during intervention?** | **Frequency/ Duration** | **Requirements of participants** |
| --- | --- | --- | --- | --- | --- | --- | --- |
| Aftyka et al. [26] | Digital imagery sharing | Nursing Staff | To support parent-infant bonding through visual connection to infants during the COVID pandemic. Parents provided with photographs and videos of their infant electronically during the COVID pandemic | Individual | Yes | Average 5 photographs a day/ short films (10-30 seconds) sent twice weekly | Receiving of electronically delivered images/ films |
| Archibald. S[27] | Father’s support group | Female member of NICU psychology team and male member of chaplaincy | To emotional and social support through peer support | Group | No | Offered twice per month (one during lunchtime 1pm-2pm, and the other after working hours 6pm-7pm)  Study ran for 9 months | Optional attendance of group. No commitment required. |
| Ardal et al.[28] | Parent buddy matching by language/ culture | Veteran parents who attended a training program.  Unit social worker acted as a consultant for the buddy during the relationship. | To provide social and emotional support through cultural buddy matching | Individual | No | Frequency of contact ranged from 5 to more than 20 conversations over a period of 1-12 months | Either face to face of telephone interactions with parent buddy. Open-ended conversations. Some also emailed in addition to this. Duration and frequency as requested by mother. |
| Beleninik et al. [29] | Music Therapy | Music Therapist | To support mother-infant bonding by decreasing levels of parental stress, increasing well-being, improving coping in parents until 6 months corrected age | Individual | Yes | 2 sessions per week during NICU stay and 2 across a 3 months time line at home. | Participation in music therapy sessions. This could be through music making or by being present whilst therapist creates music. |
| Bracht et al. [30] | Parent education and support program | Staff and Veteran Parents | Enhance family integrated care, to promote mothers confidence and competency in care of their infants | Small group education with individual teaching as needed | No | Education sessions were daily and scheduled for one week however participants could attend multiple times across their stay.  Frequency of attendance and duration of groups not recorded. | Daily educational sessions. Provide care for infants at least 8 hours daily and participate in medical rounds |
| Corrigan et al. [31] | Music Therapy | Music Therapist | To support mother-infant bonding in the NICU through decreasing parental anxiety and improving well-being | Individual | Yes | 2 heartbeat-music interventions (recorded maternal lullaby and heartbeat for infants and recorded infant heartbeat and preferred music for mothers). Music for mothers to be used as they wished. | Infant’s heartbeat recorded, parents to choose a song meaningful to them, parents told to use the recording as they wished. |
| Dahan et al. [32] | Peer support group | Veteran NICU parents | Peer support to decrease isolation, gain confidence in parental role, and be a source of parent education | Individual | No | 10 week study period, 1hr per week | Attendance of at least 1 of 10 ‘parent meetings’ |
| Van Dokkum et al[33] | Music Therapy | Music Therapy | To improve parent wellbeing and bereavement support, parent-infant bonding. | Individual | Yes | Frequency and duration of ongoing psychotherapeutic music therapy not specified. Recordings once created continue to be visited in sessions for the duration of stay. | Attendance of music therapy sessions.  Choice of ‘song of kin’ (song with meaning to family). |
| Epstein et al. [34] | Music Therapy | Music Therapist | To support mother-infant bonding through decreasing maternal anxiety and supporting well-being | Individual | Yes | 2 sessions per week during NICU stay and 2 across a 3 months time line at home.  Total number ranged from 2 NICU with 0 at home to 27 NICU with 6 at home. | Participation in music therapy sessions. This could be through music making or by being present whilst therapist creates music. |
| Ettenberger et al. [35] | Music Therapy during Kangaroo care | Music Therapist | To decrease parental anxiety and improve parent-infant bonding. | Individual | Yes | 2 sessions weekly until hospital discharge. Average length of sessions 19 minutes (range 10-40 minutes) | Completion of at least two therapy sessions. Singing songs with music therapist that were important to family’s culture during kangaroo care. Alternatively, parents could opt for receptive sessions where music therapist improvised music on guitar. |
| Feeley et al[36] | ‘PMAC’ (Promoting Mother’s Ability to Communicate) Program: a)how to identify and reduce anxiety b)education sessions on sensitive interaction | a)Cognitive behaviour therapist  b)graduate nurse and psychologist | To reduce parental anxiety and enhance sensitivity | Individual | No | 6 x 1hr teaching sessions. 1-2 sessions per week.  Final session at home 2-4 weeks after discharge. | Attendance of 6 sessions. Reading of booklet prior to session. Learning exercises to be applied to their own infant. Observation of interaction with infant videoed for discussion with professional. |
| Ghetti et al[37] | Music Therapy | Music Therapist | To support parent-infant bonding by enhancing ‘emotional closeness’ to the infant | Individual | Yes | 2 sessions per week during NICU stay and 2 across a 3 months time line at home. | Participation in music therapy sessions. This could be through music making or by being present whilst therapist creates music. |
| Grieb et al[38] | Mindfulness | Video and audio recordings provided by researcher | To promote mothers’ mental health and well being | Individual | No | 2 sets of materials one for during first weeks of admission and the other 1-3 weeks later: 20 minute introductory video, 4 mindfulness topics with audio recordings 5/10 minutes long.  Trial ran for 7 months. | Recordings to be used as participants wished. |
| Haslbeck et al.[39] | Music Therapy | Music Therapist | To facilitate bonding and attachment through supporting parent social-emotional wellbeing and parental empowerment | Individual | Yes | 20 minute session 2-3 times per week | Parent to hold infants throughout session where music is provided by music therapist. Parents encouraged to respond to infant’s cues as well as hum and speak. |
| Helmer et al.[40] | Early Collaborative Intervention | NIDCAP trained staff | To improve maternal mental health by providing positive experiences after pre-term birth. Aim to reduce maternal anxiety. | Individual | Yes | 3 sessions: 2 in the NICU and one when infant is full-term in family home. | Participation in infants care assisted by an ‘EACI’ provider. Summary of notes provided for parents after sessions with photographs taken during sessions to assist parents to recall details of session. |
| Hurst I. [41] | Parent support group, one to one support and telephone calls. | Veteran parent and nurse | Parental emotional support, information sharing and facilitating parents in baby’s care. | Group/ Individual | No | Weekly parent group, one to one parent support and telephone support | Attendance or involvement in group or individual support. |
| Joyce et al.[42] | ‘Beads of Courage’ | Clinical staff trained in the program | To support families who are coping with serious or life-threatening illness by decreasing distress and finding meaning in illness. | Individual | No | Study period 2 years, details of each infant admission not reported. | To add beads to string to represent milestones, treatments, or procedures. |
| Kobus et al.[43] | Music Therapy | Music Therapist | To promote engagement with infant and support parent wellbeing | Individual | Yes | Twice weekly from second week of life until discharge.  Duration range 10-50 minutes. | Optional attendance in music therapy with infant listening to music created by therapist and ‘sansula’ instrument. |
| Loewy et al. [44] | Music Therapy | Music Therapist | To ‘repair a broken or fragmented sense of self-protection, build agency and resilience, and integrate the memory of traumatic event | Individual | Yes | Duration of NICU stay, frequency not stated. | Initial meeting with therapist,  Engagement in music therapy with infant. This may include singing, sharing songs that have meaning to the family or playing instruments. |
| Marshall et al. [45] | Mindfulness | Staff trained in mindfulness based stress reduction | To reduce parent stress during infant admission to NICU. | Individual | No | Enrolled 2 weeks after birth, 1hour training session until discharge.  Asked to practice techniques for duration of infants admission to NICU. (relaxing sighs and calming phrases daily, meditation twice a week). | Attendance of training session in a private room away from bedside of infant that included 3 core techniques from mindfulness practice. Links provided for downloadable mindfulness programs and access to meditations on an iPad kept on the NICU. |
| Mendelson et al. [46] | Mindfulness | Introduced by research assistant | Enhance attention, awareness and compassion for self and others improving mood anxiety and self-regulation. | Individual | No | Use of video as frequently as parents wished. Followed up 2 weeks after baseline. | Viewing of introductory video on mindfulness and audio recorded practices. |
| Mouradian et al.[47] | Art therapy based scrapbooking group | Occupational therapist and Art therapist assisted by social workers and a nurse | Aims to reduce parent stress and anxiety | Group | No | Data collected of a 5-month period during weekly 2hr sessions. | Attendance of drop-in scrapbooking group. Parents could leave at any point. |
| Nottage. S [48] | Non-Medical support services (Social worker, Chaplain, Family support group, sibling playgroup, family support centre, parent-parent support) | Multidisciplinary Team | Parent support/ wellbeing | Group and Individual | No | Minimum 3 week period. Duration and frequency of interventions varied dependant on which was accessed. | Optional access to variety of support services available. Multiple services could be accessed. |
| Ormston et al.[49] | Music Therapy | Music therapist | To support parents with their anticipatory grief and create positive experiences tosupport and bonding during NICU stay and end of life. | Individual | Yes | Weekly sessions | Optional attendance of music therapy with or without infant. |
| Parker et al [50] | Counselling/psychotherapy | Qualified counsellor/ psychotherapist in a sister role on the NICU | To process experiences/ events and the emotions connected with them in a safe contained space to support with coping with the NICU admission | Individual | No | Not specified | Parents to enter into diagloue with a trained counsellor about their experiences and emotions whist in a private space. |
| Pearson et al.[51] | Parent sharing circle | Facilitator skilled in working with parents and families of premature infants | To promote positive parenting in the NICU to support parent confidence and reduce feelings of loss of control | Group | No | 90 minutes | Attendance of group with other NICU parents. In the group parents are encouraged to share pregnancy and birth experience, learning about developmental challenges, development of understanding of infants skills and capabilities. Understanding of infant care plan, and direction to available resources. |
| Preyde M and Ardal. F[52] | Parent buddying | Trained veteran parents of preterm infants | Peer support to increase social and emotional support for parents who experience preterm birth | Individual and group | No | 16 week duration | Primarily telephone support provided by veteran parent. Group also available |
| Russel et al [53] | Journalling with some prompts in journal | Journal provided whilst mother on postnatal ward | To improve parent experiences of the NICU and reduce stress. | Individual | No | Minimum of 2 weeks and maximum of 4 weeks. | To journal as frequently as parents choose. Option to share topics they wrote about with researcher. |
| Schwarz et al.[54] | ‘Rush Keepsakes’ Scrapbooking group and family photo shoots. | NICU staff nurses | To document and celebrate steps towards NICU discharge and created mementos to ‘reconsolidate the loss of the ideal and the real infant’. | Group/ family | No- Scrapbooking  Yes- photoshoot | Data from 6 months of the program:  Weekly 2hr scrapbooking session (alternates weekdays and weekends)  Holiday photoshoots | Voluntary attendance of scrapbooking group or photographs taken of family together.  For scrapbooking parents are invited to bring photographs, foot/ hand prints, locks of hair, monitor leads, nappies and other caregiving items. Dummies are attached to books via a ribbon. |
| Scott.Z and Archbald.S [55] | Fathers support group | Female member of NICU psychology team and male member of chaplaincy | To promote the importance of fathers, social support, provide space to share their experiences. | Group/ individual if attendance low | No | Data from support groups captured between January and March 2019. | Attendance of fathers group. |
| Shoemark. H[56] | Music Therapy informed parent education program | Music Therapist | Promotion of parent-infant interaction and understanding of infant and parent expressive capability. | Individual | No | One off session lasting up to 1 hour. | Attendance of one parent session away from cot with information booklet to take away. |
| Simon et al.[57] | CBT based intervention group | Psychology fellows and student therapists | To reduce symptoms of PTSD through building trusted relationships and contact with others with similar experiences | Group | No | Not reported | Attendance of group-based therapy |
| Turner et al.[58] | Group parent support and education | Child and adolescent psychiatrist and neurodevelopmental physiotherapist | To reduce parent stress through providing supportive space | Group | No | Not reported  (group commenced 24 months prior to data collection) | Attendance of group |
| De Vasconcelos et al. [59] | Mothers support group | Multidisciplinary team (nurses, social workers, psychologists and students in nursing, biology and social communication) | To provide social and emotional support during NICU admission | Group | No | Not reported | Optional attendance of support group |
| Wharton et al. [60] | Group trauma-focused cognitive behaviour therapy | Psychology fellows and student therapists | Processing of traumatic events | Group | No | 6 group sessions | Writing of trauma narratives and sharing them with the group |
| White et al. [61] | ‘Art therapy’ | Artist | To improve parental well-being | Individual | Yes | Not reported | Art session with artist using a range of art materials including SLR camera |

App.3 **Table of Analysis**

| **Author** | **Sekhon domain (X)** |  |  |  |  |  |  |
| --- | --- | --- | --- | --- | --- | --- | --- |
| Aftyka et al. [26] | **Affective Attitude** | **Burden** | **Ethicality** | **Intervention Coherence** | **Opportunity costs** | **Perceived Effectiveness** | **Self-Efficacy** |
| Archibald. S[27] |  | X |  |  |  | X | X |
| Ardal et al.[28] |  |  |  |  | X | X |  |
| Beleninik et al. [29] | X | X | X | X |  | X |  |
| Bracht et al. [30] | X | X |  |  |  | X | X |
| Corrigan et al. [31] |  | X |  | X | X | X |  |
| Dahan et al. [32] |  | X |  |  |  | X | X |
| Van Dokkum et al[33] |  |  |  |  |  | X |  |
| Epstein et al. [34] |  | X |  |  |  | X | X |
| Ettenberger et al. [35] | X | X |  |  |  | X | X |
| Feeley et al[36] |  |  |  |  |  | X |  |
| Ghetti et al[37] |  |  |  |  |  | X |  |
| Grieb et al[38] | X |  |  | X | X | X | X |
| Haslbeck et al.[39] |  |  |  | X | X | X |  |
| Helmer et al.[40] | X | X |  | X | X | X | X |
| Hurst I. [41] | X |  |  | X |  | X | X |
| Joyce et al.[42] | X | X |  |  | X | X |  |
| Kobus et al.[43] |  | X |  |  |  | X |  |
| Landry |  |  |  |  |  |  |  |
| Loewy et al. [44] |  |  |  |  | X | X |  |
| Marshall et al. [45] |  |  |  |  |  | X |  |
| Mendelson et al. [46] |  |  |  |  | X | X |  |
| Mouradian et al.[47] |  |  |  |  |  | X |  |
| Nottage. S [48] |  |  |  |  |  | X |  |
| Ormston et al.[49] | X |  |  |  |  | X |  |
| Parker et al [50] |  |  |  |  |  | X |  |
| Pearson et al.[51] | X |  |  |  | X | X |  |
| Preyde M and Ardal. F[52] | X |  |  |  |  | X |  |
| Russel et al [53] |  |  |  |  |  | X |  |
| Schwarz et al.[54] |  |  |  |  |  | X |  |
| Scott.Z and Archbald.S [55] |  |  |  |  |  | X |  |
| Shoemark. H[56] |  |  |  |  | X | X |  |
| Simon et al.[57] | X | X | X |  | X | X | X |
| Turner et al.[58] |  |  |  |  |  | X |  |
| De Vasconcelos et al. [59] |  |  |  |  |  | X |  |
| Wharton et al. [60] |  | X |  |  |  | X |  |
| White et al. [61] | X | X |  |  |  | X | X |

App 4. Risk of Bias assessments

1. CASP qualitative studies checklist

|  | Was there a clear statement of aims of the research? | Is a qualitative methodology appropriate? | Was the research design appropriate to address the aims of the research? | Was the recruitment strategy appropriate to the aims of the research? | Was the data collected in a way that addressed the research issue? | Has the relationship between researcher and participants been adequately considered? | Have ethical issues been taken into consideration? | Was the data analysis sufficiently rigorous? | Is there a clear statement of findings? | How valuable is the research? |
| --- | --- | --- | --- | --- | --- | --- | --- | --- | --- | --- |
| Aftyka el al | Y | Y | Y | Y | Y | Can’t tell | Y | Y | Y | Researcher previously known to participants in psychologist role. Results are transferable to the wider neonatal population and considerations made for neonatal practice. |
| Ardal et al. | Y | Y | Y | Y | Y | Y | Y | Y | Y | Transferrable data to wider settings where any parent-buddy system is in place |
| Bieleninik et al | Y | Y | Y | Y | Y | N | Can’t tell | Y | Y | Small sample size results unlikely to be transferrable; music therapists provided sessions and interviewed parents on their experience |
| Bracht et al. | Y | Y | Y | Can’t tell | Y | Can’t tell | Can’t tell | Y | Y | Relationship between researchers and participants unclear. Potential pressure to respond as asked to complete questionnaire immediately after group session. Position of influence does not seem to have been noted however coding was conducted by 2 researchers independently. |
| Dahan. S et al | Y | Y | Y | Y | Y | Can’t tell | Can’t tell | Y | Y | Relationship between researchers and participants unclear. Potential pressure to respond as asked to complete questionnaire immediately after group session. Position of influence does not seem to have been noted however coding was conducted by 2 researchers independently. |
| Dantas J. M et al | Y | Y | Y | Y | Y | N | Y | Y | Can’t tell | Researchers own potential for bias not examined. Only one researcher analysed data. Limitations of the study not discussed. |
| Van Dokkum N et al | Y | Y | Y | Y | Y | Can’t tell | Y | Y | Y | Transferrable data however small sample with potential selection bias and bias of review from authors. |
| Epstein et al | Y | Y | Y | Y | Y | Y | Y | Y | Y | Small sample therefore unlikely to be transferrable however clear stance of researcher noted and considered. |
| Ghetti et al | Y | Y | Y | Y | Y | N | Can’t Tell | Y | Y | Music Therapist interviews own participants that have had music therapy with them. Although provided with opportunity to give negative feedback this has been the only means of considering the implication of this. Minimal mention of ethical considerations |
| Grieb. S et al | Y | Y | Y | Y | Y | Y | Y | Can’t tell | Y | Transferable data. Consideration as to the discrepancy of data collection points has not been noted or discussed. Strong attempt to remove researcher bias addressed throughout. |
| Harmon. R et al. | Y | Can’t tell | Can’t tell | Y | Y | Y | N | Can’t tell | Y | Good sample of data however considerations for the vulnerability of the participants does not appear to have been made. Analysis process unclear. |
| Haslbeck. F et al. | Y | Y | Y | Can’t tell | Y | Y | Y | Y | Y | Transferrable data although small sample size. Recruitment is not clearly documented with no reason stated for why only 6 of 13 families participated in the interviews. |
| Helmer. S et al. | Y | Y | Y | Y | Y | Y | Y | Y | Y | High quality study however full details of participants and their infants not provided therefore difficult to determine how transferrable data is to the wider neonatal population. |
| Hurst | Y | Can’t tell | Y | Y | Y | Can’t tell | Y | N | Y | Low response rate (23%) as postal survey with 25% of surveys undeliverable. Details of responses appear potentially selective however challenges and areas for improvement are noted. |
| Joyce et al. | Y | Y | Y | Y | Y | Y | Y | Y | Y | High quality study with consideration on transferability of an intervention previously used in oncology to neonatal population |
| Parker et al. | Y | Y | Y | Y | Y | Can’t tell | Y | N | Y | Demographics of mothers not provided therefore judgment cannot be made if this group is representative of population. |
| Pearson | Y | Y | Y | Y | Y | Can’t tell | Y | Y | Y | Researcher bias or stance not mentioned. Large sample of evidence however participant characteristics not recorded and therefore difficult to access transferability or confounding factors. |
| Scott. A and Archibald | Y | Y | Y | Can’t tell | Y | N | Can’t tell | Y | Y | Relationship between researchers and participants unclear. Demographic not collected and therefore unable to consider how transferrable this data is to the wider population. |
| Shoemark. H | Y | Y | Y | Y | Y | Y | Y | Y | Y | Small scale study of mothers only, predominantly Australian. Therefore not easily transferrable to other countries. However researcher bias and stance has been carefully considered. |
| Turner M et al. | Y | Y | Y | Can’t tell | Y | Can’t tell | Y | Y | Y | Father's declined participation, this is not considered further however researchers note that support for fathers requires further investigation. |
| De Vasconcelos et al. | Y | Y | Y | Y | Y | Can’t tell | Y | Y | Y | Reasonable sample size, demographics of mothers not documented and therefore not easily transferable. |
| White. M et al | Can’t tell | Y | Y | Y | Y | Can’t tell | Y | Can’t tell | Y | No demographic data or explanation of analysis of questionnaire. Not comment on stance of researcher or potetial bias. Data for reasons for declining to participate not documented or fully considered. |

1. JBI Critical appraisal checklists: Case reports

|  | Were patient’s demographic characteristics clearly described? | Was the patient’s history clearly described and presented as a timeline? | Was the current clinical condition of the patient on presentation clearly described? | Were diagnostic tests or assessment methods and the results clearly described | Was the intervention (s) or treatment procedure (s) clearly described? | Was the post-intervention clinical condition clearly described? | Were adverse events (harms) or unanticipated evented identified and described? | Does the case report provide takeaway lessons? | Comments |
| --- | --- | --- | --- | --- | --- | --- | --- | --- | --- |
| Lowey et al. | Y | Y | Y | Y | Y | Y | N | Y | Researcher bias not considered |
| Ormston. K et al | Y | Y | Y | Y | Y | Y | N | Y | Researcher’s bias not considered |
| Schwarz et al | N | N | N | N | Y | Y | Y | Y | No documentation of participant demographics. No comment on researcher’s stance/ bias |

1. ROB2


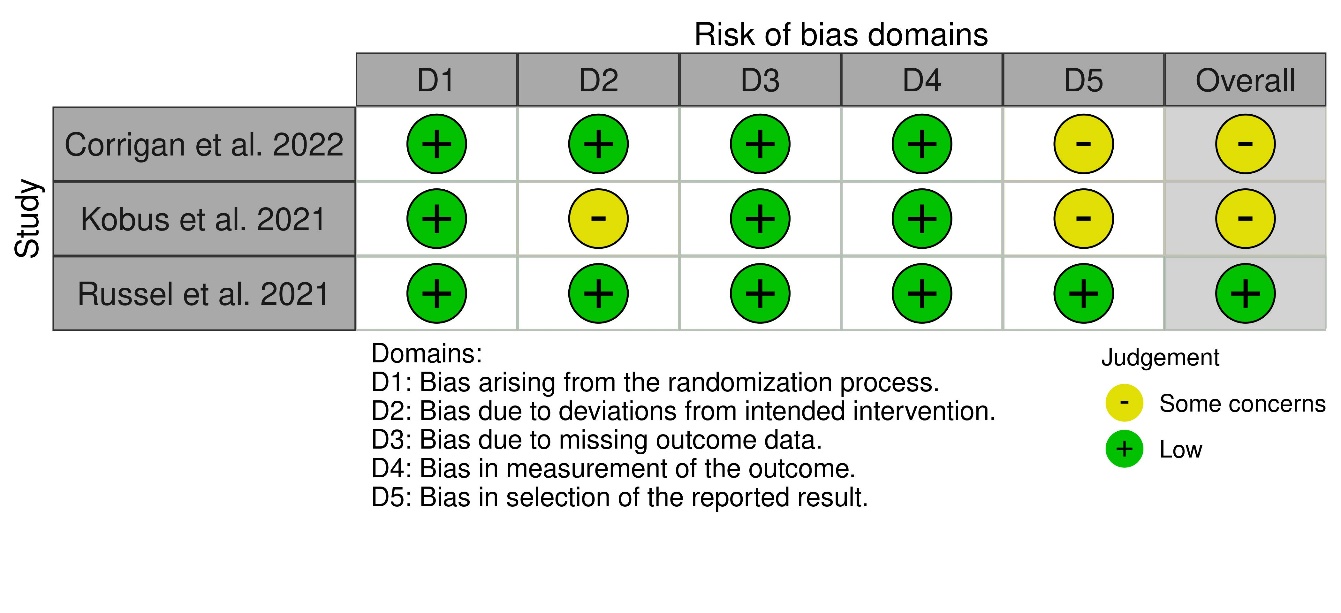


ROBINS-I


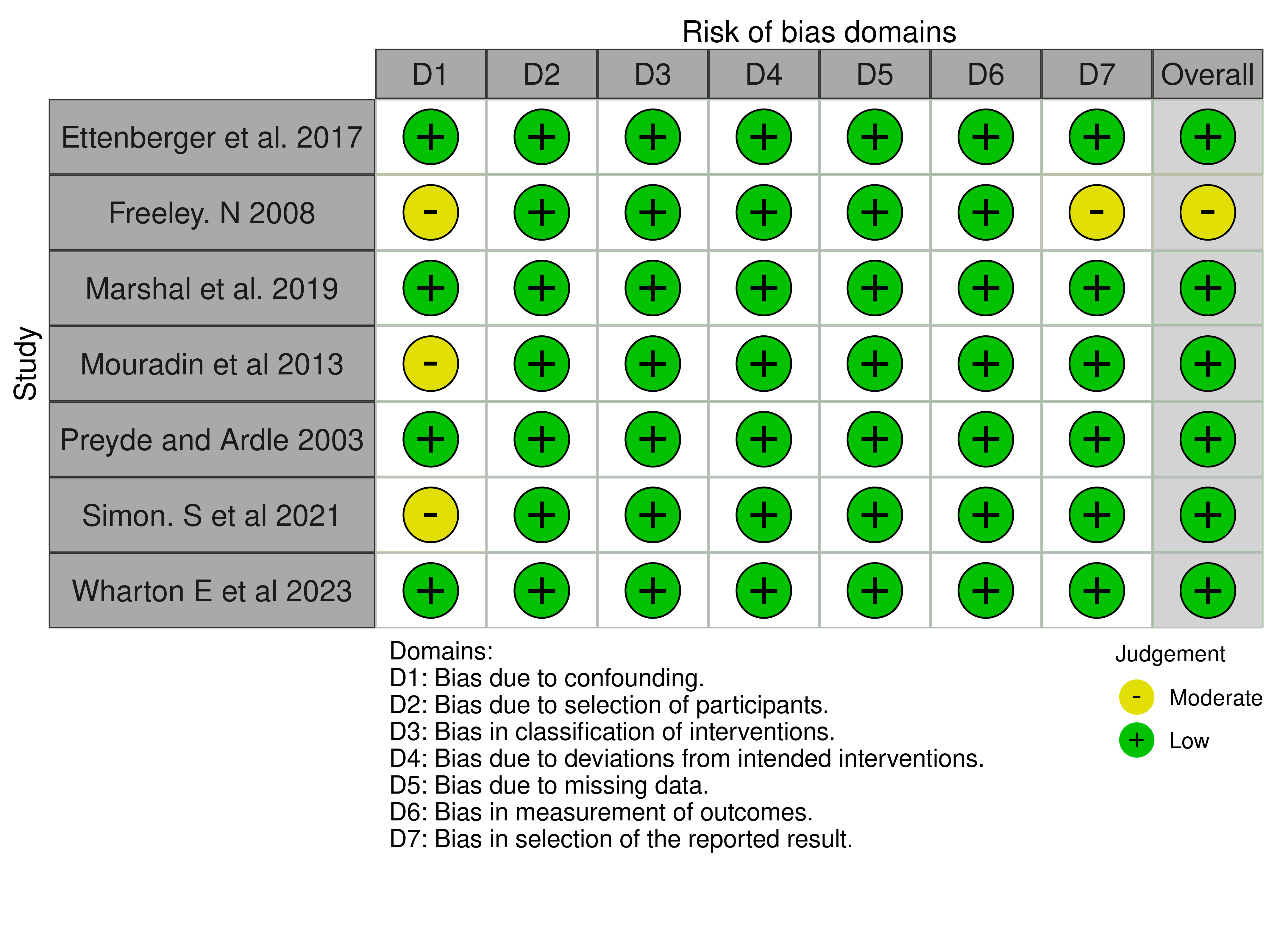


App .5 Prisma checklist

| **Section and Topic** | **Item #** | **Checklist item** | **Location where item is reported** |
| --- | --- | --- | --- |
| **TITLE** | | |  |
| Title | 1 | Identify the report as a systematic review. | Reported on PROSPERO  (CRD42023491268) |
| **ABSTRACT** | | |  |
| Abstract | 2 | See the PRISMA 2020 for Abstracts checklist. | At start of submission |
| **INTRODUCTION** | | |  |
| Rationale | 3 | Describe the rationale for the review in the context of existing knowledge. | Introduction |
| Objectives | 4 | Provide an explicit statement of the objective(s) or question(s) the review addresses. | Introduction |
| **METHODS** | | |  |
| Eligibility criteria | 5 | Specify the inclusion and exclusion criteria for the review and how studies were grouped for the syntheses. | Section 2.2 |
| Information sources | 6 | Specify all databases, registers, websites, organisations, reference lists and other sources searched or consulted to identify studies. Specify the date when each source was last searched or consulted. | Section 2.1 |
| Search strategy | 7 | Present the full search strategies for all databases, registers and websites, including any filters and limits used. | Section 2.1 plus Example in appendix |
| Selection process | 8 | Specify the methods used to decide whether a study met the inclusion criteria of the review, including how many reviewers screened each record and each report retrieved, whether they worked independently, and if applicable, details of automation tools used in the process. | Section 2.2 |
| Data collection process | 9 | Specify the methods used to collect data from reports, including how many reviewers collected data from each report, whether they worked independently, any processes for obtaining or confirming data from study investigators, and if applicable, details of automation tools used in the process. | Section 2.4 |
| Data items | 10a | List and define all outcomes for which data were sought. Specify whether all results that were compatible with each outcome domain in each study were sought (e.g. for all measures, time points, analyses), and if not, the methods used to decide which results to collect. | Section 2.4 |
|  | 10b | List and define all other variables for which data were sought (e.g. participant and intervention characteristics, funding sources). Describe any assumptions made about any missing or unclear information. | Section 2.4/ Table 2 |
| Study risk of bias assessment | 11 | Specify the methods used to assess risk of bias in the included studies, including details of the tool(s) used, how many reviewers assessed each study and whether they worked independently, and if applicable, details of automation tools used in the process. | Section 2.3 |
| Effect measures | 12 | Specify for each outcome the effect measure(s) (e.g. risk ratio, mean difference) used in the synthesis or presentation of results. | Not applicable |
| Synthesis methods | 13a | Describe the processes used to decide which studies were eligible for each synthesis (e.g. tabulating the study intervention characteristics and comparing against the planned groups for each synthesis (item #5)). | Section 4/ Figure 2 |
|  | 13b | Describe any methods required to prepare the data for presentation or synthesis, such as handling of missing summary statistics, or data conversions. | Not applicable |
|  | 13c | Describe any methods used to tabulate or visually display results of individual studies and syntheses. | Section 4/ Table 2. |
|  | 13d | Describe any methods used to synthesize results and provide a rationale for the choice(s). If meta-analysis was performed, describe the model(s), method(s) to identify the presence and extent of statistical heterogeneity, and software package(s) used. | Meta analysis not performed |
|  | 13e | Describe any methods used to explore possible causes of heterogeneity among study results (e.g. subgroup analysis, meta-regression). | Discussed section 4 |
|  | 13f | Describe any sensitivity analyses conducted to assess robustness of the synthesized results. | Not applicable |
| Reporting bias assessment | 14 | Describe any methods used to assess risk of bias due to missing results in a synthesis (arising from reporting biases). | Not applicable |
| Certainty assessment | 15 | Describe any methods used to assess certainty (or confidence) in the body of evidence for an outcome. | Not applicable |
| **RESULTS** | | |  |
| Study selection | 16a | Describe the results of the search and selection process, from the number of records identified in the search to the number of studies included in the review, ideally using a flow diagram. | Figure 3 |
|  | 16b | Cite studies that might appear to meet the inclusion criteria, but which were excluded, and explain why they were excluded. | Figure 3 (do I include excluded articles in an appendix to cite?) |
| Study characteristics | 17 | Cite each included study and present its characteristics. | Table 2 |
| Risk of bias in studies | 18 | Present assessments of risk of bias for each included study. | Appendix |
| Results of individual studies | 19 | For all outcomes, present, for each study: (a) summary statistics for each group (where appropriate) and (b) an effect estimate and its precision (e.g. confidence/credible interval), ideally using structured tables or plots. | Not applicable |
| Results of syntheses | 20a | For each synthesis, briefly summarise the characteristics and risk of bias among contributing studies. | Table 2 and appendix |
|  | 20b | Present results of all statistical syntheses conducted. If meta-analysis was done, present for each the summary estimate and its precision (e.g. confidence/credible interval) and measures of statistical heterogeneity. If comparing groups, describe the direction of the effect. | Not applicable |
|  | 20c | Present results of all investigations of possible causes of heterogeneity among study results. | Discussed section 4 |
|  | 20d | Present results of all sensitivity analyses conducted to assess the robustness of the synthesized results. | Not applicable |
| Reporting biases | 21 | Present assessments of risk of bias due to missing results (arising from reporting biases) for each synthesis assessed. | Not applicable |
| Certainty of evidence | 22 | Present assessments of certainty (or confidence) in the body of evidence for each outcome assessed. | Not applicable |
| **DISCUSSION** | | |  |
| Discussion | 23a | Provide a general interpretation of the results in the context of other evidence. | Section 4 |
|  | 23b | Discuss any limitations of the evidence included in the review. | Section 5 |
|  | 23c | Discuss any limitations of the review processes used. | Section 5 |
|  | 23d | Discuss implications of the results for practice, policy, and future research. | Section 6/7 |
| **OTHER INFORMATION** | | |  |
| Registration and protocol | 24a | Provide registration information for the review, including register name and registration number, or state that the review was not registered. | Section 2.1 |
|  | 24b | Indicate where the review protocol can be accessed, or state that a protocol was not prepared. | Section 2.1 |
|  | 24c | Describe and explain any amendments to information provided at registration or in the protocol. | none |
| Support | 25 | Describe sources of financial or non-financial support for the review, and the role of the funders or sponsors in the review. | Section 8 |
| Competing interests | 26 | Declare any competing interests of review authors. | Section 5.1 |
| Availability of data, code and other materials | 27 | Report which of the following are publicly available and where they can be found: template data collection forms; data extracted from included studies; data used for all analyses; analytic code; any other materials used in the review. | Appendix referenced throughout |
